# Response to regarding the case image article entitled “A case of angle‐closure glaucoma caused by spontaneous lens dislocation” by Kondo et al.

**DOI:** 10.1002/ccr3.8855

**Published:** 2024-04-30

**Authors:** Keisuke Kondo, Hiroki Isono

**Affiliations:** ^1^ Department of General Medicine, Institute of Biomedical Science The University of Tokushima Graduate school Tokushimashi Tokushima Japan; ^2^ Department of General Medicine HITO Medical Center Shikokuchuoshi Ehime Japan

## Abstract

The occurrence of acute angle closure with posterior displacement of the lens is rare compared with that of anterior dislocation; however, several studies have reported its occurrence.

## CONSENT

Written informed consent was obtained from the patient to publish this report in accordance with the journal's patient consent policy.

Thank you for the valuable comments of our clinical image. Generally, anterior crystalline lens dislocation usually leads to angle‐closure glaucoma, while posterior crystalline lens dislocation causes ocular inflammation and visual loss. Meanwhile, this case presents glaucoma due to posterior lens dislocation.

The occurrence of acute angle closure with posterior displacement of the lens is rare compared with that of anterior dislocation; however, several studies have reported its occurrence. In a study of displaced lenses in 57 cases in 48 dogs, preoperatively, 73% (19/26 eyes) of the eyes with anterior luxations had secondary glaucoma compared to 43% (10/23 eyes) with subluxations and 38% (3/8 eyes) with posterior luxations.^1^ In the series, of the 79 eyes with posterior intraocular lens dislocation, 12 (15.2%) had glaucoma.^2^ A report from China showed that of 106 cases of traumatic lens dislocation, 26 were posterior dislocations, of which 22 (84.6%) developed glaucoma.^3^

Presently, despite the posterior dislocation, the intraocular pressure measured immediately after transferring at the new hospital was 46 mmHg in the left eye (normal value: 10–21 mmHg). Even with posterior lens dislocation, if glaucoma or inflammation develops, lens removal is indicated.^4^ The patient underwent left vitrectomy due to a diagnosis of glaucoma attack caused by posterior lens dislocation. Moreover, the intraocular pressure was 8 mmHg on postoperative day 1 and remained at 8–17 mmHg.

The mechanism of glaucoma caused by posterior dislocation is suggested to be pupillary block glaucoma attributable to vitreous herniation into the anterior chamber.^5^ Further studies are needed to elucidate the mechanism.

Thereafter, we believe angle‐closure glaucoma is reasonable.

## AUTHOR CONTRIBUTIONS

**Keisuke Kondo:** Conceptualization; data curation; formal analysis; funding acquisition; investigation; methodology; project administration; resources; software; supervision; validation; visualization; writing – original draft; writing – review and editing. **Hiroki Isono:** Conceptualization; data curation; formal analysis; funding acquisition; investigation; methodology; visualization; writing – original draft; writing – review and editing.

## FUNDING INFORMATION

All authors have read the article and approved submission. We have no potential conflicts of interest related to this article. This study was not funded. All authors have reviewed and agreed with the content of this article.

## Data Availability

Data openly available in a public repository that issues datasets with DOIs.
